# Effects of Repeated X-Radiation of the Whole Body on the Development of Tumours in Rats Due to Feeding p-Dimethylaminoazobenzene

**DOI:** 10.1038/bjc.1949.63

**Published:** 1949-12

**Authors:** Cornelia Hoch-Ligeti

## Abstract

**Images:**


### REFERENCES.

### EFFECTS OF REPEATED X-RADIATION OF THE WHOLE BODY ON THE DEVELOPMENT OF TUMOURS IN RATS DUE TO FEEDING *p*-DIMETHYLAMINOAZOBENZENE.

CORNELIA HOCH-LIGETI.

*From the Cancer Research Department, London Hospital Medical College, London, E. 1.*

Received for publication October 15, 1949.

THE effect of X-radiation in producing malignant growth in man was found soon after X-rays were introduced into general use in medicine. In 1909 eleven cases were reported in England (Rowntree, 1909). The tumours occurred after repeated exposures over a long period and they were preceded by dermatitis. They appeared on the parts of the skin directly exposed to the rays, and it was therefore concluded that the radiation absorbed in superficial layers was responsible for these changes. In animal experiments Bloch (1924) produced epitheliomata of the rabbit's ear with large doses of X-rays only, and X-rays were found to provoke malignant changes when applied to inflamed tissue in rabbits (Lacassagne and Vinzent, 1929 ; Lacassagne, 1933 ; Burrows, Mayneord and Roberts, 1937 ; Burrows and Clarkson, 1943). In combination with other carcinogenic agents, e.g. tar (Castiglioni, 1927) or carcinogenic hydrocarbons (Mayneord and Parsons, 1937), X-rays accelerated the appearance and the growth of tumours in mice. Although in these combinations both agents were acting on the skin, it was considered that the effect of X-rays was partly direct, in stimulating the cells involved in cancer production, and partly indirect, in breaking down the defence mechanism of the body. Russ, Chambers, Scott and Mottram (1919)

concluded as a result of their experiments on the effect of small doses of X-rays on the blood count and on the resistance to transplanted tumours in rats, and from evidence produced by Murphy and Morton (1915) and Murphy and Taylor (1918), that X-rays, apart from their direct action on tissue cells have two indirect actions: "(*a*) large doses of X-rays, by destroying the immune conditions, will favour the growth of tumours, and (*b*) small doses, by producing immune conditions, will help to overcome the tumour."

In order to find out whether irradiation has an indirect effect on the development of tumours, experiments on the combined effect of irradiation and a carcinogenic compound not acting on the same organ seemed of interest. The study of the influence of total body irradiation on the development of hepatic tumours, which occur with great regularity in rats fed with *p*-dimethylaminoazobenzene seemed suitable for this purpose. It was intended to irradiate with small doses, but opinions differ as to the amount of radiation that should be considered small, particularly where it is applied repeatedly over a long period of time, e.g. about half the expected lifetime of the experimental animal. In the present exploratory experiment, both penetrating and soft X-rays were used in doses which appeared reasonably small. The dosages were calculated so that the skin of the back should receive about the same amount of radiation with the soft as with the penetrating rays. The liver, however, which is the site of tumours produced by feeding *p*-dimethylaminoazobenzene, would receive with hard rays about the same amount as the skin, but with soft rays hardly any radiation at all. It was thought that under these conditions a direct and an indirect action of the radiation before and during the establishment of a tumour might be distinguished.

### EXPERIMENTAL.

Forty male and 10 female Wistar rats, weighing about 100 g. at the beginning of the experiment, were given a semi-synthetic diet, in which 53 per cent of the calories were supplied by starch and sugar, 17 per cent by casein, and 30 per cent by lard. Salt mixture and cod-liver oil were mixed with the food; greens were given once a week, and 2 to 3 g. of bread (80–85 per cent extraction) daily. *p*-dimethylaminoazobenzene was added to the food in a concentration of 0·07 per cent; the diet was prepared in quantities sufficient for about 10 days. Food and water were given *ad lib.* It had been found in earlier experiments that on this regime 90 to 100 per cent of the rats developed tumours within 6 to 10 months. In the present series 10 male rats which served as controls received no other treatment. Twenty male rats were treated with soft radiation, and 10 male and 10 female rats were given penetrating X-rays. The irradiations were begun on the same day as the feeding of *p*-dimethylaminoazobenzene, and they were made in exactly the same manner with hard and soft radiation. At the start each rat received 10 r once weekly. The physical factors of the penetrating radiation were: 180 kV, 0·75 mm. copper, 1 mm. aluminium filter (H.V.L. 1·0 mm. copper), and focal distance 30 cm. The rats received 10 r in the liver, i.e. on the ventral surface of the body. Taking the thickness of the rat as 5 cm. the dorsal surface received some 13 r at each irradiation. With the soft radiation the 10 r were calculated with respect to the dorsal surface of the body, and the liver (ventral surface) received only about 0·3 r per irradiation. Factors for the soft radiation: 45 kV, no added filter, H.V.L. 0·33 mm. aluminium, and focal distance 20 cm. Each rat was irradiated individually, in a wooden box in the case of the hard, and in a celluloid tube in the case of the soft radiation. During the course of weekly irradiations the rats receiving hard radiation lost weight considerably; those receiving soft radiation merely stopped growing. The intervals between the irradiations were then lengthened to 2 weeks, and all the rats resumed approximately normal growth. This fortnightly irradiation was kept up during the greater part of the experiment, but intervals of 3 and 4 weeks were also employed. Over the whole experimental period of 76 weeks the rats were irradiated 7 times at intervals of 1 week, 16 times at intervals of 2 weeks, 4 times at 3-weekly and 4 times at 4-weekly intervals. Thus the rats treated with hard radiation received on the skin of the back about 400 r, in the liver 310 r; the volume doses (calculated for 250 g. rats) were about 100,000 g. $\times$ *r*. The corresponding doses for the rats treated with soft radiation were: 310 r in the skin of the back, 10 r in the liver, and a volume dose of about 24,000 g. $\times$ r.

Two rats in each irradiated group and one control rat died during the first three weeks of the experiment and are not considered further.

### RESULTS.

No palpable tumours occurred up to the 8th month. All rats were therefore subjected to laparotomy under ether anaesthesia and the livers inspected. Three of the control and 4 of the hard irradiated rats showed macroscopic tumours. Two irradiated rats had cirrhosis of the liver. No macroscopic changes were observed in the livers of the rats treated with soft X-rays. From the 8th month onward tumours were palpable in some of the control and hard irradiated rats. The experiment was terminated after 76 weeks. At post-mortem the organs were weighed and fixed in formol-alcohol for histological investigation.

*Effect on tumour formation.*

Table I summarizes the data concerning the occurrence and the rate of growth of the tumours. Large (2 to 10 g.) and very large (up to 50 g.) tumours have

TABLE I.—*Number and Size of Liver Tumours in Irradiated and Control Rats fed* p-*dimethylaminoazobenzene.*

| | Number of rats. | Large and very large tumours. | Small tumours. | Microscopical tumours or suspicious changes. | No tumours. |
|---|---|---|---|---|---|
| Control, no irradiation | 9 | 5 | 3 | 1 | 0 |
| Soft irradiation | 18 | 1 | 3 | 7 | 7 |
| Hard irradiation | 18 | 7 | 4 | 4 | 3 |

been grouped together, the macroscopically visible tumours up to pea size form a second group, and tumours of microscopic size or histological changes suggestive of commencing tumour formation in a third; livers devoid of neoplastic change form the fourth group. It is evident that irradiation with soft X-rays delayed and to some extent prevented the formation of tumours. Seventy-seven per cent of these rats had either normal livers or livers showing only incipient proliferative change. No tumour of other origin was found.

In the control group all animals had hepatic tumours. In one rat a pituitary tumour was found in addition (Fig. 1). Microscopically this tumour was cystic, resembling a haemangioma ; it is impossible to say whether it was due to the *p*-dimethylaminoazobenzene.

In the group receiving hard X-rays the number of animals with very small tumours and those not developing tumours was increased as compared with the controls, but the small number of rats and the circumstance that rats of both sex were included in this group does not permit any definite conclusions. In two of these rats (both males) cutaneous tumours occurred. In one of them two primary cutaneous tumours developed, one being a slowly growing epithelioma of the back, whilst the other, which appeared near the hind leg about 3 months later, grew rapidly and consisted of solid columns of non-keratinizing epithelial cells ; microscopically it was quite different from the epithelioma (Fig. 4, 5). No hepatic tumour was found in this rat. The second rat developed a strongly keratinizing epithelioma near the right axilla. Underlying this tumour hypertrophic and cystic glandular tissue was found. The liver of this rat had several small tumours. No cutaneous tumour has ever arisen spontaneously in any of our stock rats.

Histologically the tumours in the liver were mostly hepatomas and non-cystic and cystic cholangiomas. Hepatomas and cholangiomas were often found in the same liver. No histological differences could be observed between the tumours which developed in the irradiated and in the control group, but in the healthy parts of the livers of irradiated rats there were in several cases groups of cells which were enlarged, their cytoplasm more darkly stained and their nuclei rather dark and bulbous. In many of the livers from the hard X-rayed animals an increase of the connective tissue and cirrhotic changes were more pronounced than in the controls. The livers of most of the rats irradiated with soft X-rays showed fatty infiltration and some patches where the cells were of the signet ring type. In many cases the Kupffer cells were prominent and filled with pigment (Fig. 2).

*Effects on other organs.*

The changes in the weights of the organs in the three groups of rats are summarized in Table II.

The adrenals in animals of both irradiated groups were enlarged, but this change was statistically significant only in the case of those rats irradiated with soft rays. Whether the difference in enlargement of the adrenals in the two irradiated groups is real is difficult to decide, as only the male rats of the hard irradiated group could be used for this comparison ; female rats have always larger adrenals than males. In previous experiments (Hoch-Ligeti, 1947, 1948) on the effect of the administration of small doses of penetrating and soft X-rays on healthy rats it was found that the increases in adrenal weights of male rats were significant in both cases.

The adrenals of the rats receiving the soft X-rays showed microscopically an increase of the cortex, mostly of the zona reticularis, but also of the

EXPLANATION OF PLATES.

FIG. 1.—Tumour of the pituitary in a control rat fed *p*-dimethylaminoazobenzene.
FIG. 2.—Kupffer cells filled with pigment in the liver of a rat irradiated with soft X-rays. (× 180.)
FIG. 3.—Multiple cysts in the adrenal cortex of a rat irradiated with hard X-rays. (× 65.)
FIGS. 4 and 5.—Cutaneous tumours in a rat irradiated with hard X-rays. 4 = epithelioma, 5 = columns of non-keratinizing epithelial cells. (× 95.)

1

Hoch-Ligeti.

2

3

4

5

fasciculata. In many cases large amounts of pigment were deposited in the reticularis. The cells of the zona fasciculata were larger, pale staining, and sometimes contained large amounts of fatty or lipoid material. In some cases in both fasciculata and glomerulosa nests of large, deeply staining cells were present. In some adrenals incipient cysts were found.

Very similar changes were found in the adrenals of the rats irradiated with hard X-rays, but the formation of cysts was much more pronounced. In 12 out of 18 pairs of adrenals single or multiple cysts of various sizes were found in the cortex (Fig. 3).

Similar cysts, sometimes visible to the naked eye as small red spots, were observed in the adrenals of the control rats, but the other changes seen in the irradiated animals were not found.

In both irradiated groups the weight of the pituitaries was increased, but this increase was too small to be significant statistically with the small number of animals.

The weights of the other organs did not differ in the irradiated and control groups. In particular there was no decrease in the weight of the spleen, thymus or gonads of the irradiated rats. No characteristic change could be demonstrated in the appearance of the thymus of these rats in routine histological examination, nor were any signs of lymphatic atrophy found in the spleens; on the contrary, in many cases the spleen of the soft-irradiated rats showed cellular haloes around enlarged Malpighian bodies, suggesting hypertrophy of the lymphoid and epithelioid elements. The testes, which appeared histologically normal in the control group, showed in 6 out of 10 hard-irradiated rats degeneration of the tubular epithelium with absent or greatly reduced spermatogenesis. Reduced spermatogenesis was found in 3 soft-irradiated rats, and testicular atrophy in one. In the female rats irradiated with hard X-rays the ovaries contained numerous big luteinized follicles ; Graafian follicles were absent, except in one ovary. Two uteri showed cystic hypertrophy of the dilated glands.

### DISCUSSION.

It has been established (Packard, 1932 ; Langendorff and Reuss, 1933) that the biological effect of ionizing radiation is independent of the wave-length. The different effect of soft and hard X-rays found in the present work may be due, therefore, either to a difference in the volume doses received by the two groups of rats or to differences in penetration, i.e. to a difference in the organs directly radiated. If it is assumed that a quantitative difference in the volume dose is the determining factor, it would follow that a certain system or systems involved in the mechanism or tumour development are activated by the dose delivered by soft radiation, but may be damaged in their function by the dose delivered by the hard radiation. If the difference in penetration is the determining factor, it is remarkable that in the experiment with hard radiation in which the liver received 310 r of direct radiation, no definite protection against carcinogenesis by *p*-dimethylaminoazobenzene was found, yet with soft radiation where only a negligible quantity of rays could have affected the liver directly, the livers were protected against the effect of *p*-dimethylaminoazobenzene. It appears, therefore, that radiation may have some indirect effect on tumour formation.

It has been suggested that the state of the reticulo-endothelial system is a decisive factor in tumour development. The vast amount of work and discussion bearing on this question has been reviewed by Stern and Willheim (1943). Relevant work was published recently by Stern (1948) and Davidsohn and Stern (1949). The findings in the present experiment are not contradictory to the assumption that the reticulo-endothelial system is affected by the radiation. Dougherty, White and Chase (1944) and Dougherty and White (1945) consider that the lymphoid organs and the state of immunity is dependent on the secretions of the adrenals and the pituitary. In the work here presented soft radiation produced a significant increase in the weight of the adrenals and a slight increase in the weight of the pituitary. The histological changes of these adrenals are consistent with a hyperfunctional hypertrophy. The cystic changes found in the adrenal cortex of the hard-irradiated rats could be interpreted as a sign of degeneration after excessive stimulation. Changes suggestive of a direct activation of the cells of the reticulo-endothelial system are the large increase of Kupffer cells in the liver and the hypertrophy of the epithelioid elements in the spleen of the irradiated rats which did not develop tumours.

The question of an indirect effect of radiation on the body generally when X-rays are used locally to destroy tumours was reinvestigated by Kaplan and Murphy (1949). They found that in mice the dose used locally for the destruction of malignant cells might adversely affect the body defences against the development of metastases. Similar conclusions were reached by Russ, Chambers, Scott and Mottram (1919) on theoretical grounds.

Apart from one subcutaneous sarcoma which occurred after injection of very large doses of *p*-dimethylaminoazobenzene (Kinosita, 1937), this substance has never been shown to provoke cutaneous tumours, nor are X-rays carcinogenic for the rat skin unless previously irritated either by chemical carcinogens or inflammation. The development of skin tumours in the present experiment suggests that the feeding of *p*-dimethylaminoazobenzene sensitizes the skin to the carcinogenic action of X-rays. Experiments on a larger scale are in progress to extend the observations reported.

### SUMMARY.

Groups of rats on a diet containing *p*-dimethylaminoazobenzene underwent total body irradiation with small doses of soft or penetrating X-rays.

Whilst all non-irradiated rats receiving *p*-dimethylaminoazobenzene developed hepatic tumours, the development of tumours was prevented, or their rate of growth retarded, in rats treated with soft radiation.

The growth of hepatic tumours was slightly retarded in rats treated with penetrating rays. Skin tumours developed in 2 of these rats.

The adrenals of rats receiving soft X-rays were significantly enlarged.

The author wishes to thank Mr. R. Oliver and Mr. M. Cohen for the calculation of the physical factors and for their help with the irradiation, Dr. H. Ney for helpful discussions, and Mr. K. Goodall and Miss D. Connell for their skilful technical assistance. The author is indebted to the British Empire Cancer Campaign for a grant held during the investigation.

### REFERENCES.

Bloch, B.—(1924) *Schweiz. med. Wschr.*, **54**, 857.
Burrows, H., and Clarkson, J. R.—(1943) *Brit. J. Radiol.*, **16**, 383.
*Idem*, Mayneord, W. V., and Roberts, J. E.—(1937) *Proc. Roy. Soc.*, B, **123**, 213.
Castiglioni, G.—(1927) *Soc. ital. di diol. speriment*, **2**, 16.
(Quoted from Selig, M. G., and Cooper, Z. K.—(1933) *Amer. J. Cancer*, **17**, 589.)
Davidsohn, I., and Stern, K.—(1949) *Cancer Research*, **9**, 462.
Dougherty, T. F., and White, A.—(1945) *Amer. J. Anat.*, **77**, 81.
*Iidem* and Chase, J. H.—(1944) *Proc. Soc. exp. Biol., N.Y.*, **56**, 28.
Hoch-Ligeti, C.—(1947) *Ann. Rep. Brit. Emp. Cancer Campgn.*, **25**, 93.—(1948) *Ibid.*, **26**, 121.
Kaplan, H. S., and Murphy, E. D.—(1949) *J. nat. Cancer Inst.*, **9**, 407.
Kinosita, R.—(1937) *Trans. Jap. path. Soc.*, **27**, 665.
Lacassagne, A.—(1933) *C. R. Soc. Biol., Paris*, **112**, 562.
*Idem* and Vinzent, R.—(1929) *Ibid.*, **100**, 249.
Langendorff, H. and M., and Reuss, A.—(1933) *Strahlentherapie*, **46**, 289.
Mayneord, W. V., and Parsons, L. D.—(1937) *J. Path. Bact.*, **45**, 35.
Murphy, J. B., and Morton, J. J.—(1915) *J. exp. Med.*, **22**, 204.
*Idem* and Taylor, D.—(1918) *Ibid.*, **28**, 1.
Packard, C.—(1932) *Amer. J. Cancer*, **16**, 1217.
Rowntree, C. W.—(1909) *Lancet*, i, 821.
Russ, S., Chambers, H., Scott, G. M., and Mottram, J. C.—(1919) *Ibid.*, i, 692.
Stern, K.—(1948) *Proc. Soc. exp. Biol., N.Y.*, **67**, 315.
*Idem* and Willheim, R.—(1943) 'The Biochemistry of Malignant Tumors.' Brooklyn: Chemical Publishing Co., Inc.


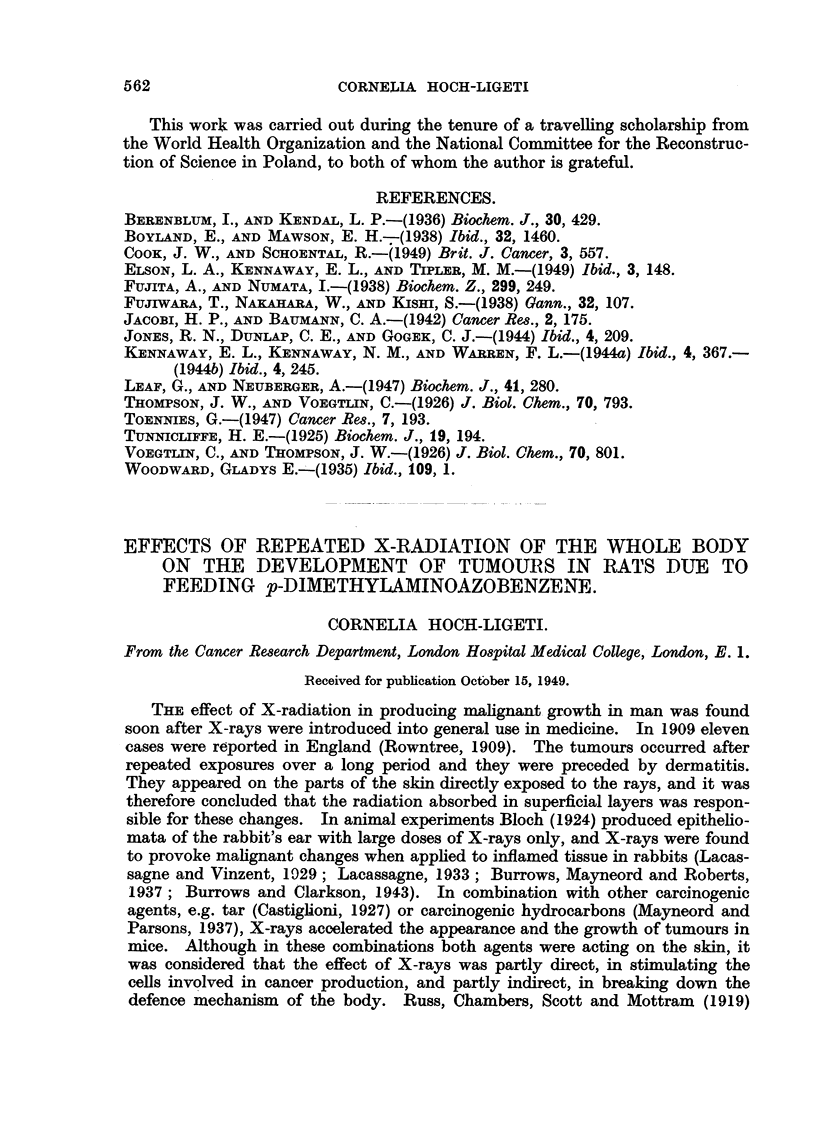

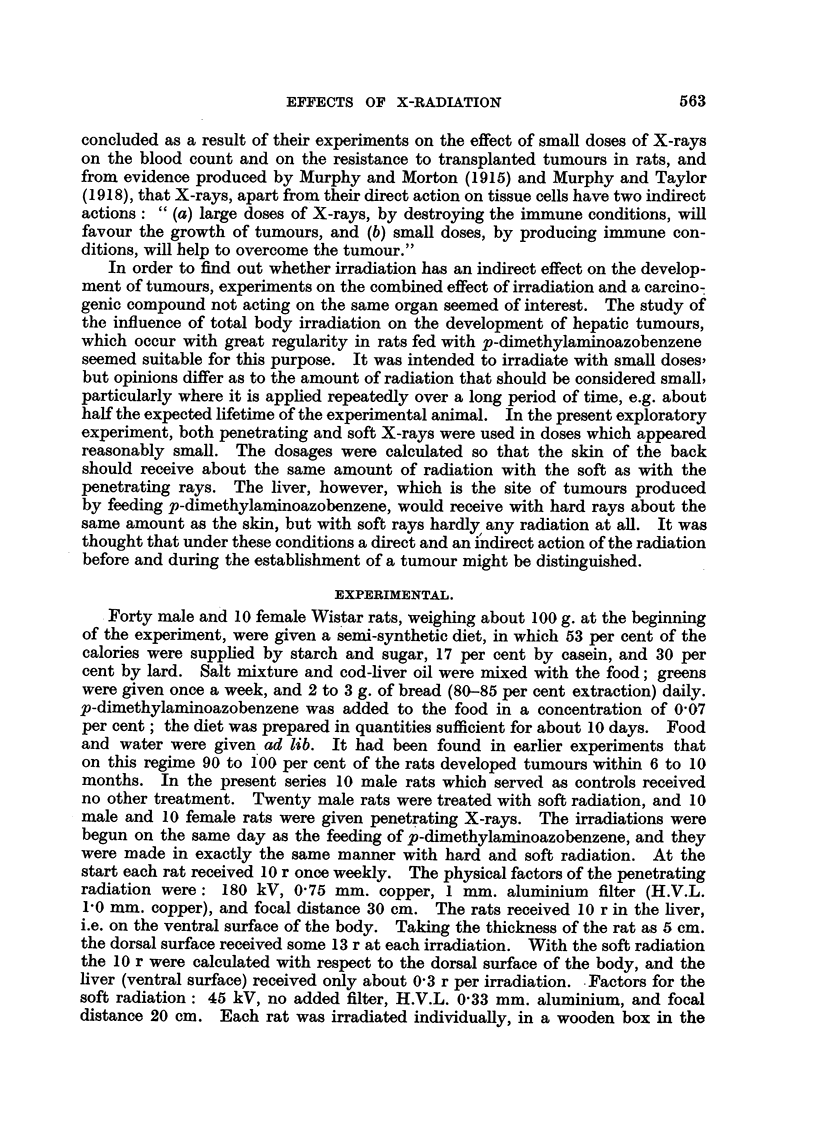

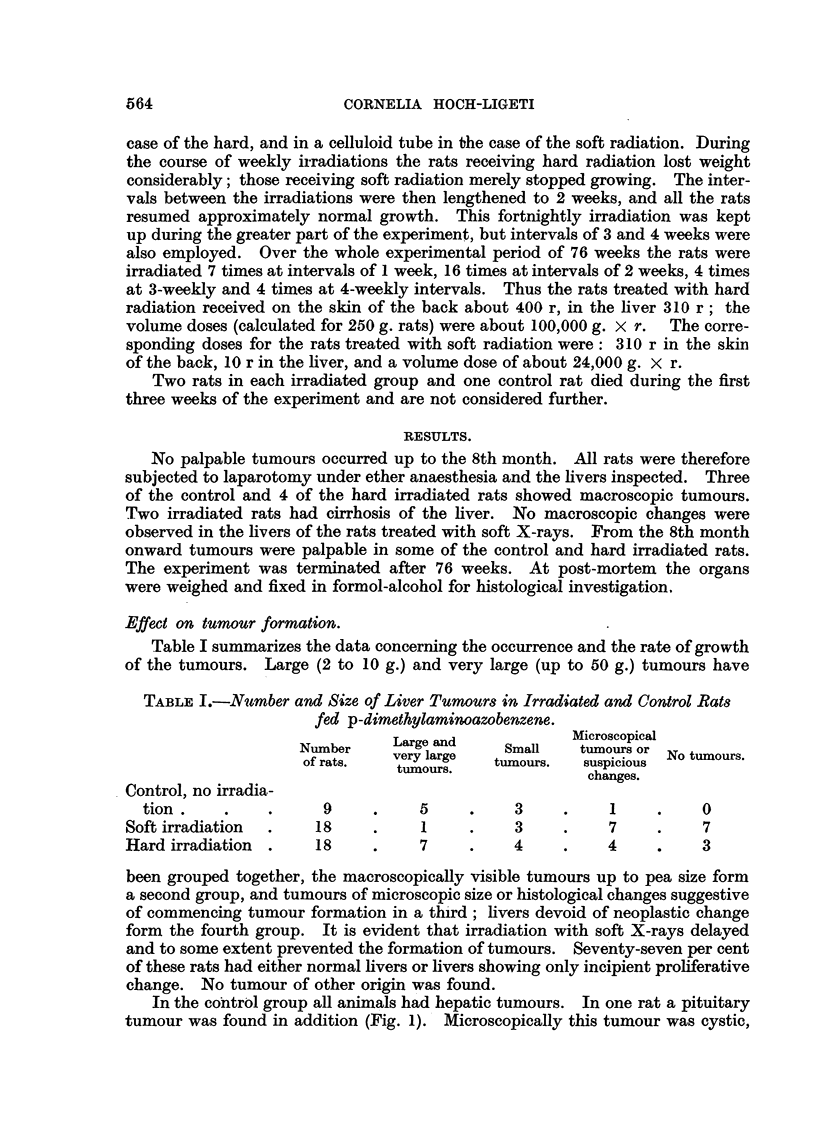

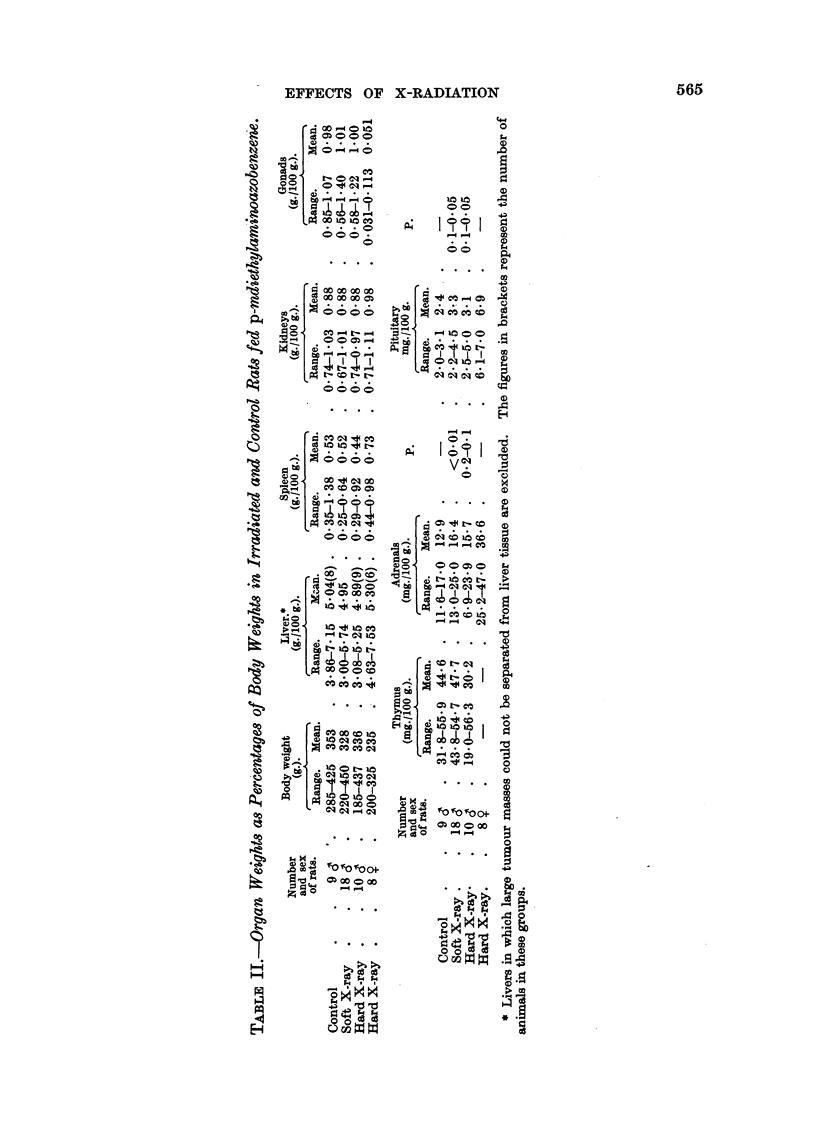

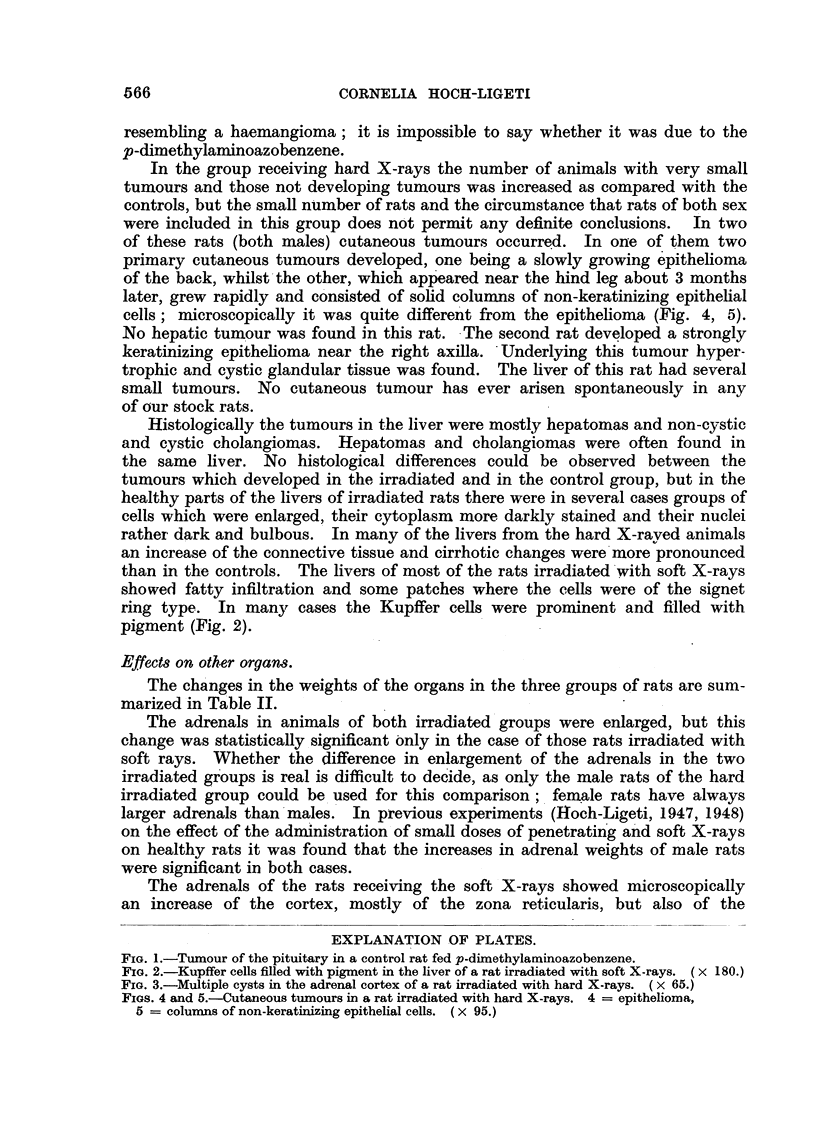

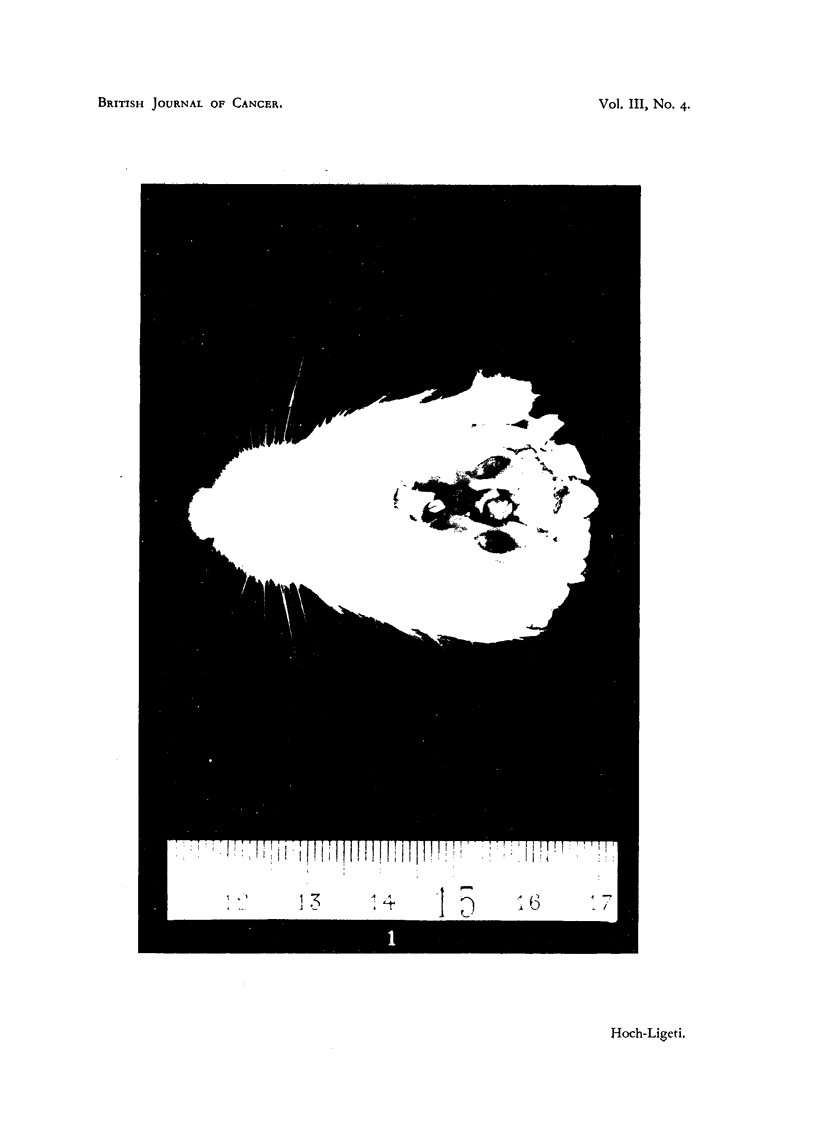

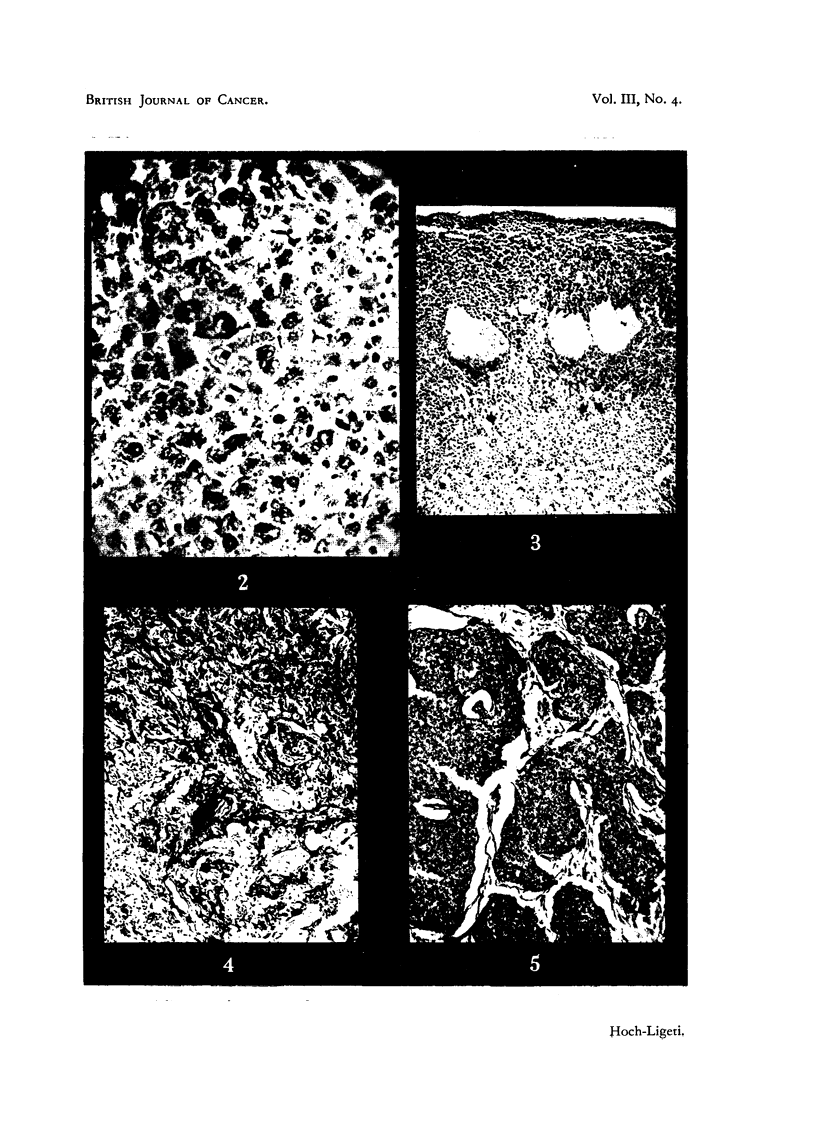

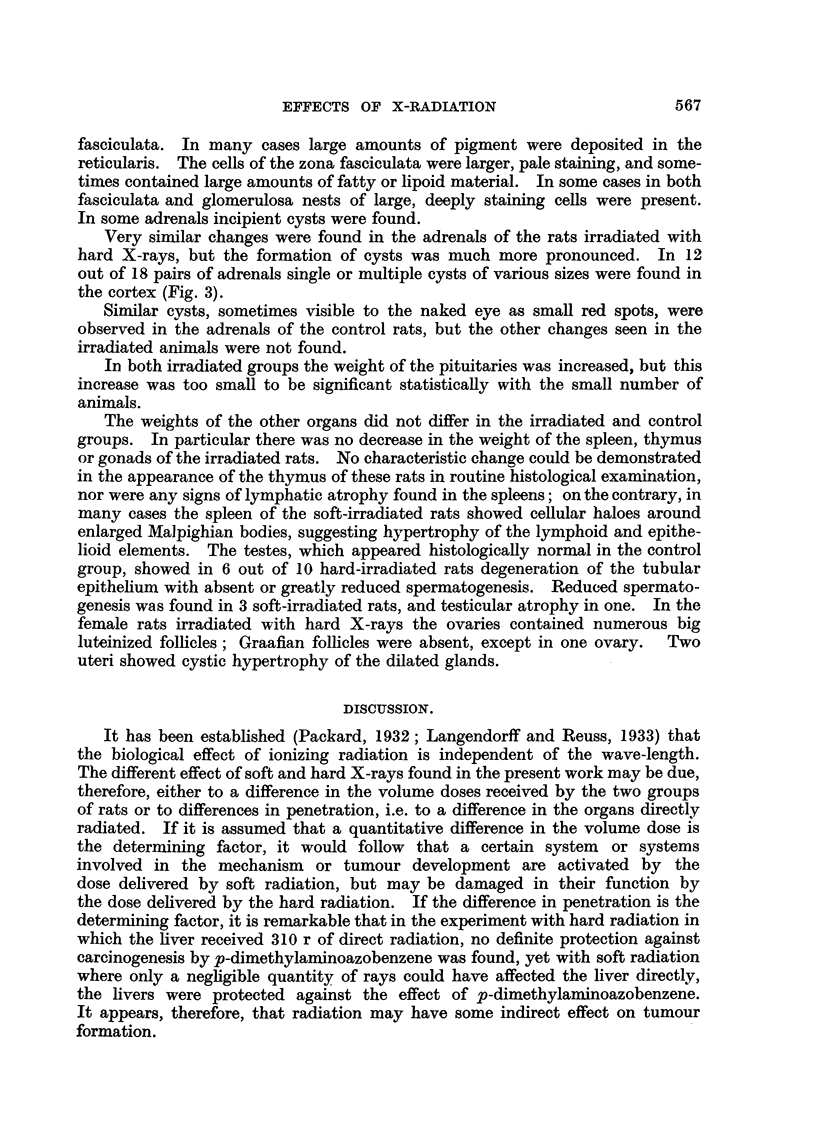

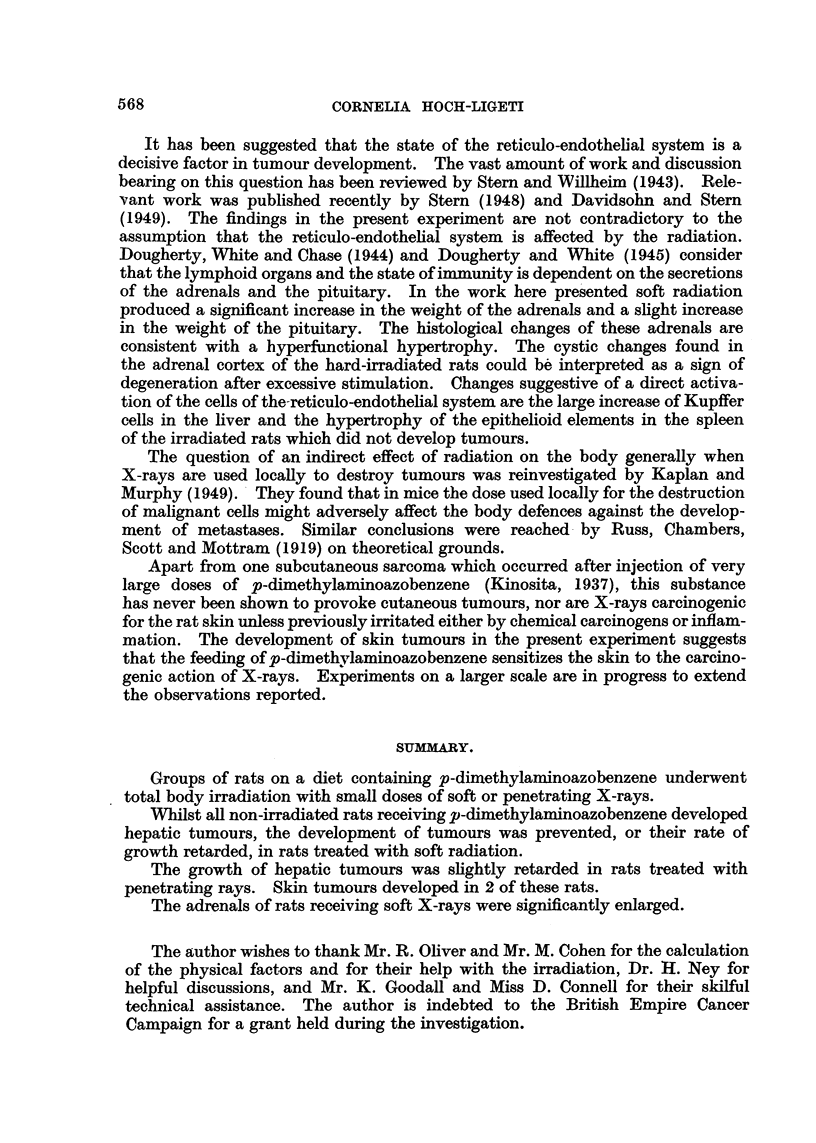

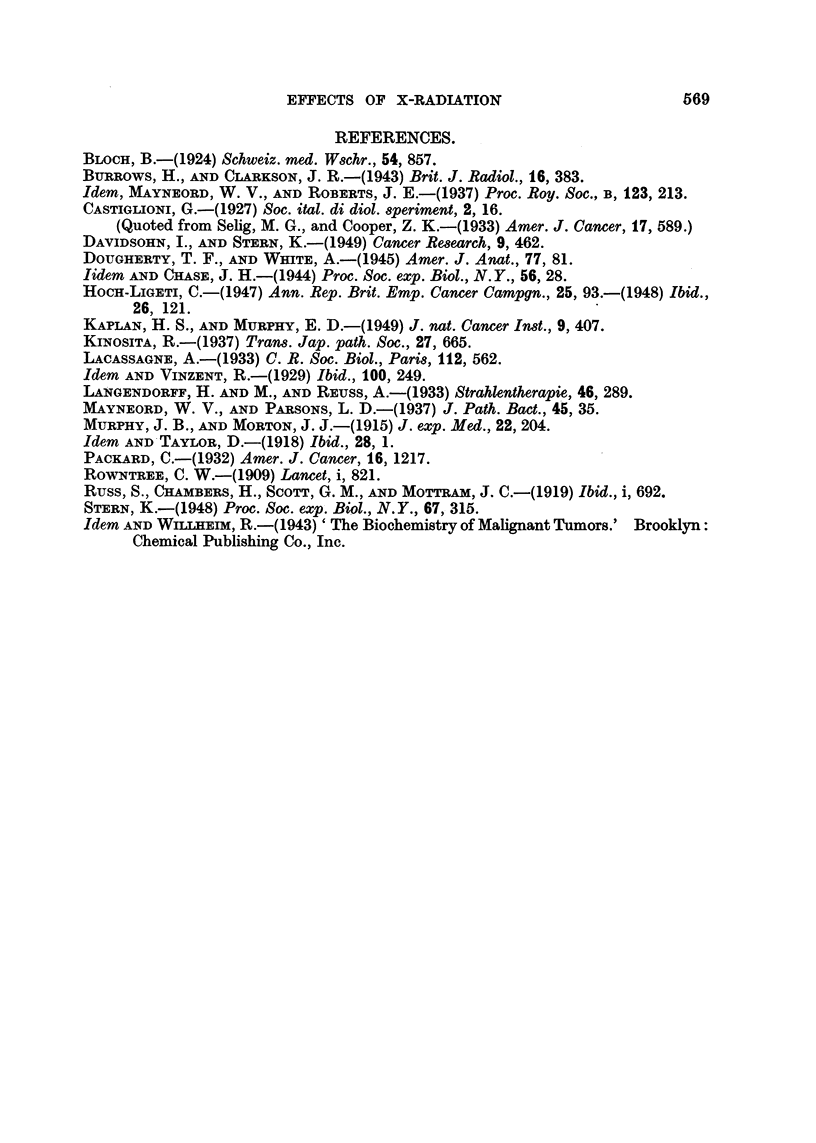


## References

[OCR_00494] KAPLAN H. S. (1949). Lesions of the gastric mucosa in Strong strain NHO mice.. J Natl Cancer Inst.

